# Risk and Loss Aversion and Attitude to COVID and Vaccines in Anxious Individuals

**DOI:** 10.5334/cpsy.115

**Published:** 2025-02-07

**Authors:** Filippo Ferrari, Jesse Alexander, Peggy Seriès

**Affiliations:** 1 Institute for Adaptive and Neural Computation, School of Informatics, University of Edinburgh, United Kingdom

**Keywords:** Anxiety, Risk aversion, Loss aversion, COVID-19, Computational Psychiatry

## Abstract

Anxious individuals are known to show impaired decision-making in economic gambling task and in everyday life decisions. This impairment can be due to aversion to uncertainty about outcomes (risk aversion) and/or aversion to negative outcomes (loss aversion). We investigate how non-clinical individuals with high levels of Generalised Anxiety Disorder symptoms (GAD) (N = 54) behave compared to less anxious subjects (N = 61) in a gambling decision-making task delivered online and designed to separate the distinct influences of risk and loss aversion on decision-making. By modelling subjects’ choices using computational models derived from Prospect Theory and fitted using Hierarchical Bayesian methods, we estimate individual levels of risk and loss aversion. We also link estimates of these parameters to individual propensity to risk averse behaviours during the COVID pandemic, like wearing safer types of face masks, or completing a COVID vaccination course. We report increased loss aversion in individuals with increased level of GAD compared to less anxious individuals and no differences in risk aversion. We also report no evidence for a link between risk and loss aversion and attitudes towards COVID and vaccines, under the experimental conditions and incentive scheme studied here. These results shed new light on the interplay of anxiety and risk and loss aversion and they can provide useful directions for clinical intervention.

## 1 Introduction

Anxiety disorders have long been associated with behavioural differences in decision-making (Bishop and Gagne, 2018). Here we examine economic decisions in which two main factors are at play: risk and loss aversion. A famous behavioural economics theory called Prospect Theory (Kahneman and Tversky, 1979) describes how humans prefer sure outcomes with lower magnitude (low risk) compared to less sure outcomes with higher magnitude (high risk), which results in risk averse behaviours. Prospect Theory also describes how individuals are generally considered loss averse, i.e. humans prefer to avoid losses to acquiring gains of equivalent value, usually with a 2:1 ratio between losses and gains.

In this study we use computational models derived from Prospect Theory to quantify the influence of risk and loss aversion on human decision-making. We select a gambling task developed by Sokol-Hessner et al. (2009), and previously employed by Charpentier, Aylward, et al. (2017) in anxiety. We adapt it to be administered online on a non-clinical sample of adults. This task is particularly interesting as it has been one the first of its kind which allows to separate the influence of risk aversion (i.e., aversion to uncertainty about outcomes) and loss aversion (i.e., aversion to negative outcomes) on economic decision-making.

The impact of anxiety on risk aversion has been studied in several studies generally linking increased level of anxiety to increased risk aversion biases in decision-making tasks (Maner et al., 2007; Mueller et al., 2010; Giorgetta et al., 2012; Charpentier, Aylward, et al., 2017; see Hartley and Phelps, 2012; Phelps, Lempert, and Sokol-Hessner, 2014; Bishop and Gagne, 2018; Wake, Wormwood, and Satpute, 2020, for reviews). Despite this, results from studies employing computational models are mixed. As discussed above, Charpentier, Aylward, et al. (2017) reports increased risk aversion in anxious individuals, whereas Xu et al. (2020) reports no difference in risk aversion for low and high anxiety groups. Two recent studies (Wise et al., 2022; Pike et al., 2023) also failed to observe a relationship between anxiety and differences in risk-taking, despite using different decision-making tasks. A recent meta-analysis (Wake, Wormwood, and Satpute, 2020) on the effects of fear (and anxiety) on risk-taking reports a small to moderate effect size linking fear to increased risk aversion, but with high heterogeneity in effect sizes.

Loss aversion, on the other hand, has only been investigated in anxiety in a handful of studies. A study by Charpentier, Martino, et al. (2016) found that low trait anxiety individuals showed increased changes in loss aversion and that this increase was modulated by an emotional manipulation performed in their task. Charpentier, Aylward, et al. (2017) found that individuals with high anxiety did not show differences in loss aversion compared to the low anxiety individuals, consistent with some previous work in the literature (for a review: (Sediyama, de Castro Martins, and Martins Teodoro, 2020)). Another study by Xu et al. (2020) reports increased loss aversion and no differences in risk aversion in high anxious individuals compared to low anxious subjects.

Both risk and loss aversion are important factors in anxiety with major effects, not only on obvious economic decisions, but also on individuals’ decisions in everyday life. This is particularly true in crises situation such as observed during the COVID-19 pandemic. When the health of large populations is at stake, it is important to understand what motivates specific attitudes and behaviours toward vaccinations, preemptive measures like wearing face masks and adherence to government guidelines. For example, intention to be vaccinated was associated with more positive beliefs around COVID vaccines and reduced beliefs that these would lead to negative side effects or be unsafe (Sherman et al., 2021). A recent longitudinal study (Shou et al., 2022) showed that risk averse individuals in Australia were more likely to act in a way that would reduce their risk of contracting COVID-19. At the same time, anxious individuals showed increased avoidance in implementing changes in their actions as the pandemic developed, for example continuing to cancel family events or trips even as the pandemic eased off. The first result is also consistent with Wise et al. (2022), where it is reported that higher risk aversion is linked to higher perceived threat from the pandemic. In our study, we are interested in determining whether behaviours like receiving COVID vaccinations, following government’s guidelines, wearing more protective types of face masks are modulated by anxiety and individual propensity to taking risks and/or accepting losses. We hypothesise that individual who adopted safer attitudes and behaviours around COVID would show increased risk aversion, and that this difference is modulated by anxiety.

We also model choice and reaction data using Hierarchical Drift Diffusion Models (HDDMs) (Wiecki, Sofer, and Frank, 2013), a hierarchical Bayesian extension to a type of sequential sampling model used to model how the evidence is integrated to perform quick binary decisions. The standard model used in this study (Ratcliff, 1978) assumes that, at each trial, evidence is accumulated until it reaches on of two decision thresholds which in turn triggers a response. These types of models have been previously used in anxiety research (see Roberts and Hutcherson, 2019, for a review) and to investigate risk and decision-making (Diederich and Trueblood, 2018; Konovalov and Krajbich, 2019; Sheng et al., 2020; Molter et al., 2022).

In summary, this work has therefore several goals. First, we replicate and extend, using an online version of the same gambling task, previous work (Charpentier, Aylward, et al., 2017) linking anxiety to increased risk aversion. Second, we investigate how individual levels of anxiety as well as risk and loss aversion relate to decision-making in the context of the COVID pandemic.

## 2 Methods

### 2.1 Ethics

The study was approved by the Informatics Research Ethics Process (application number 2019/84385) of the School of Informatics, University of Edinburgh. All participants gave informed consent.

### 2.2 Participants

We recruited 163 participants based in the United Kingdom (80 with no COVID vaccinations, 83 with 2 or more doses of an approved COVID vaccine) in the months of September and October 2022 through Prolific. Participants’ data was anonymised by Prolific and further anonymised after data collection. Participants were selected from the Prolific users residing in the United Kingdom and who received either 0 or 1+ dose of an approved COVID vaccine. Participants were paid £2.67 (the task took on average 20 minutes to complete, corresponding to a pay of £8/hr) with the possibility to win up to £1 proportional to their performances during the task. This sample size was motivated by a power analysis based on the results reported by Charpentier, Aylward, et al. (2017): a total sample size of 104 participants, with 52 participants in each group, is required to detect a difference in risk aversion with a Cohen’s *d* = 0.72 using a two-tailed independent t-test at a significance level of 0.05 and a power of 0.95.

We employed exclusion criteria based on attention checks and on the participants behaviour, these are described in detail in the Supplementary Information. After applying these exclusion criteria, we conducted the analysis on 115 subjects.

### 2.3 Task

**Figure 1:**
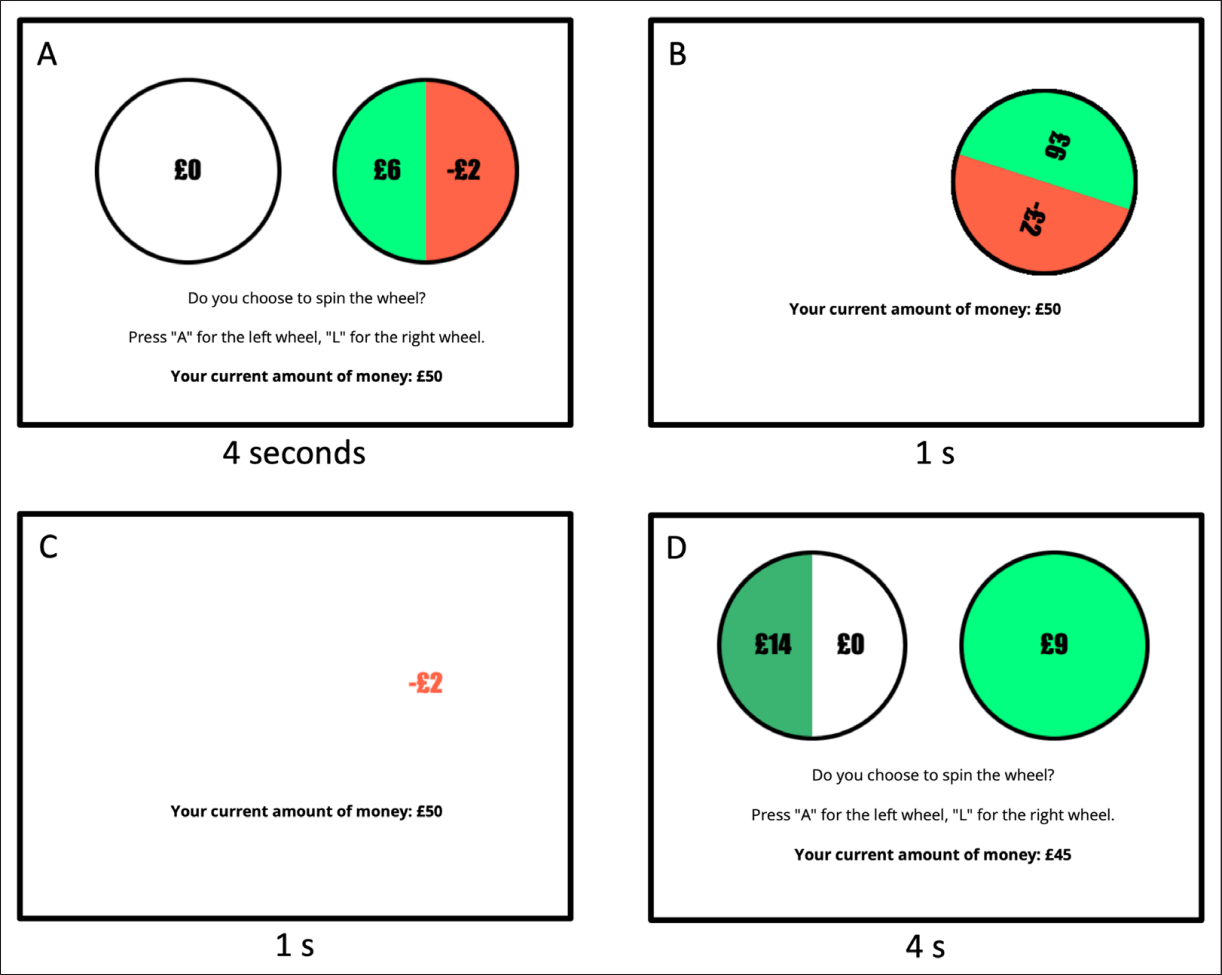
**Task structure. (A)** The frame shows a mixed gamble trial. The wheels are shown for 4s. If a participant does not make a choice within this time frame, the trial results in a time out. Once a wheel is chosen a 1s animation is shown **(B)**, followed by the amount won/lost shown for 1s **(C)**. The last frame **(D)** shows a gain-only trial, which has the same trial structure.

The task was adapted from Charpentier, Aylward, et al. (2017) and implemented using jsPsych (de Leeuw, 2015). It could only be completed using a computer. During the gambling task participants are competing in a game show where, at each trial, they are asked to make a choice between a gambling wheel and a sure option. Each option on the gambling wheel has a 50% probability to occur. Example trials are shown in Figure 1. There are two types of trials that are shown to the participants, *mixed gamble* trials and *gain-only* trials. In mixed gamble trials, one option leads to a sure option of £0, whereas the other option consists of a wheel with a 50% chance to win a monetary reward, and a 50% chance to lose some of the money accumulated so far. In gain-only trials, the sure option consists of a fixed, small amount of money, whereas the other option consist of a wheel with a 50% chance to win a larger monetary reward, and a 50% chance to win nothing. During gain-only trials, only risk aversion contributes to choosing from the safe option compared to the gambling option, while both risk and loss aversion contribute to choosing from the safe option in mixed gamble trials. At the beginning of the experiment, participants are instructed to win as much money as possible, and they are told that they will receive a bonus payment through Prolific based on their performance during the experiment. A practice section consisting of 40 trials is used to introduce the participants to the task and to adapt, through a double staircase procedure, the amounts shown at each trial to a range of expected values centered on the subject’s indifference point, i.e. the expected value corresponding to an accept rate of 50% between the gamble and the sure (or loss) option, as in previous studies (Charpentier, Martino, et al., 2016; Charpentier, Aylward, et al., 2017). Indifference points are used to determine the values of the gambles shown to the participant in the main section of the experiment. We provide details of the staircase procedure and indifference points in the Supp. Information. The main section of the experiment consists of 148 trials (98 mixed gamble trials and 50 gain-only trials in random order, the same proportion of mixed gamble/gain-only used in Charpentier et al.), in which participants have to select an option within 4s, followed by a 2s animation showing the sure/win/loss amount. Participants were instructed to use keys [A] and [L] of their keyboards to choose between options.

### 2.4 Questionnaires

At the end of the gambling task, participants were asked to complete several questionnaires. First, we administered the State Trait Anxiety Inventory (STAI) (Spielberger, 1977) to measure state and trait anxiety scores, two measures of anxiety that have widely been used in computational studies (Raymond, Steele, and Seriès, 2017), and the GAD-7 (Spitzer et al., 2006) questionnaire to assess Generalised Anxiety Disorder. Following these, participants were asked 13 questions about the COVID pandemic. Four questions asked about the subject vaccination status, the type of mask used throughout the pandemic, how anxious they were feeling at the beginning of the pandemic and how closely they followed government guidelines during the pandemic. We matched these questions with four questions asking about the subject’s future attitude towards vaccine boosters, masks, anxiety about attitude towards rules and guidelines in the case of a new surge of COVID cases. The last 5 questions asked about the subject’s general attitude towards COVID and vaccines. All these questions are rated on a Likert-scale of [1–5] and, based on the answers, we computed a COVID score where higher values corresponds to more cautious behaviour during the COVID pandemic (for example, completing the vaccination cycle, following government guidelines and wearing N95 masks). The COVID questionnaire and rating scale is reported in full in the Supp. Information.

### 2.5 Prospect Theory Models

We fitted three different models derived from Prospect Theory to the subjects’ data. The models are the same as those used in Charpentier, Aylward, et al. (2017): the first model measures both risk and loss aversion, while the other two are nested models which only measure loss and risk respectively. The models have been fitted using both maximum likelihood estimation (MLE) in MATLAB and using hierarchical Bayesian methods implemented in the hBayesDM R library (Ahn, Haines, and Zhang, 2017).

The first model (allP) captures both risk and loss aversion and is described by the following equations


1 \[u(gamble)=0.5 \times {(gain)^{\rho}+ 0.5 \times \lambda \times \left(-loss\right)^{\rho}}\]

2 \[u(sure)={{(sure)}^{\rho}}\]

3 \[p(gamble)=\frac{1}{1+{{e}^{-\mu \left[u(gamble)-u(sure)\right]}}}\]

where *u* is the subjective utility of gambling or choosing the sure option, *gain* and *loss* are the numeric values shown on the gambling wheel with losses encoded as negative numbers and *sure* is the value of the sure option. The difference between the subjective utilities is then passed through a softmax function parameterised by inverse temperature *μ* (eq.(3)); this remains the same across all models. The model has three parameters:

- *λ* represents loss aversion. When *λ* > 1, the subject weights losses more than gains and it is therefore loss averse; *λ* < 1 represents the inverse.
- *ρ* represents risk aversion. When *ρ* < 1, the subjects is risk averse, whereas with *ρ* > 1 the subject is risk seeking.
- *μ* is the inverse temperature of the action selection function where higher values of *μ* represent less stochastic decisions.

The second model (noRA) has two parameters, one for loss aversion *λ* and one for the inverse temperature *μ*:


4 \[u(gamble)=0.5\times (gain)\ +\ 0.5\ \times \lambda \times \left(-loss\right)\]

5 \[u(sure)=(sure)\]

and the third model (noLA) similarly only includes a parameter for risk aversion *ρ* and the inverse temperature *μ*:


6 \[u(gamble)=0.5\times {{(gain)}^{\rho}}+\ 0.5\times {{\left(-loss\right)}^{\rho}}\]

7 \[u(sure)={{(sure)}^{\rho}}\]

The Prospect Theory models were fitted using both maximum likelihood estimation (MLE) and hierarchical Bayesian (HB) methods. Maximum Likelihood models were fitted using MATLAB R2021b. We run constrained optimisation (fmincon with default settings) for *λ, ρ, µ* ∈ [0, ∞]. For each subject we run optimisation 20 times and we select the best set of parameters according to their negative log likelihood. Hierarchical Bayesian models were fitted using R version 3.6.3 and hBayesDM (Ahn, Haines, and Zhang, 2017) version 1.2.1 which, in turn, uses Stan (Carpenter et al., 2017) version 2.21.7. We fitted 4 chains for each model, with 2000 burn-in samples and 6000 samples (8000 samples in total). We visually inspected traces for each parameter for good mixing and checked that the Gelman–Rubin statistic \[\hat{R}\] (Gelman and Rubin, 1992) was close to 1 for all fitted parameters to test for convergence.

#### 2.5.1 Parameter Recovery

We performed extensive simulation and parameter recovery for the first model using both maximum likelihood estimation (MLE) and Hierarchical Bayesian methods (HB). We tested experimental designs comprising 148, 222 and 296 trials, with different combinations of Indifference Points and low/high anxiety parameters. For each combinations of parameters and Indifference Points(IP) we simulated 100 participants. Simulation parameters for low and high anxiety agents were taken from the existing literature (Charpentier, Aylward, et al., 2017). We report correlations using the more robust Spearman correlation coefficient, due to outliers resulting from the MLE fitting procedure. Across all combinations of parameters, fitting procedure and number of trials, recovery is successfull, with at least a Spearman *ρ* > 0.64. Parameter recovery results for 148, 222 and 296 trials and full details of the parameter recovery set up are reported in the Supp. Inf. We set the length of the experiment to 148 trials due to the similar results of the parameter recovery across different number of trials.

#### 2.5.2 Model Selection

We fitted all three models using both Maximum Likelihood Estimation and Hirarchical Bayesian methods. We compared them using the Akaike Information Criterion (AIC) and the Bayesian Information Criterion (BIC) for the parameters fitted using MLE and we compared them using Leave One Out Information Criterion (LOOIC) for the HB fitting.

#### 2.5.3 Hierarchical Modelling Specification

Previous research (Valton, Wise, and Robinson, 2020) found that effect sizes may be underestimated when assuming that all the participants of a study emerge from a single population. This is due to shrinkage effects (Gelman, Carlin, et al., 2014) common to hierarchical Bayesian models. Valton and colleagues found that accounting for hypothesised group differences in the hierarchical model specifications provides more reliable parameter estimates.

Due to our sample being recruited from a subclinical population, we will first present the results of our single prior model as we cannot rely on participants’ groups determined by a diagnosis. We will later present the results of the best split according to LOOIC scores to ease comparison with previous studies in the field (Charpentier, Aylward, et al., 2017; Xu et al., 2020) which focused on group differences in their results. Charpentier and colleagues conducted their study on a pathological anxious group meeting criteria for a GAD diagnosis with a matching group of healthy controls; while Xu and colleagues conducted their study on two groups of participants with low and high levels of STAI Trait anxiety selected from a larger pool of healthy participants.

We estimate parameters for the winning model according to six different hierarchical priors specifications (similarly to Ahn, Vasilev, et al., 2014; Aylward et al., 2019): a single prior for each parameter encompassing all participants, two separate priors for each parameter for low and high anxiety subjects according to a clinically informed cut-off of GAD-7 scores, two separate priors for each parameter for low and high anxiety subjects according to the STAI Trait median split, two separate priors for each parameter for vaccinated and unvaccinated subjects, four different priors for each parameter for the combinations of GAD-7 low/high anxiety and vaccinated/unvaccinated subjects and four different priors for each parameter for the combinations of STAI Trait low/high anxiety and vaccinated/unvaccinated subjects. Throughout the rest of this work we refer to these models as single prior, GAD-7 anxiety priors, Trait anxiety priors, vaccination priors and GAD-7/Trait anxiety and vaccination priors for convenience.

### 2.6 Hierarchical Drift Diffusion Models

Hierarchical Drift Diffusion Models (HDDMs) are a type of sequential sampling models which allow to discriminate between the different elements that are involved when making a decision. Contrary to the Prospect Theory models, HDDMs model both choice and reaction times concurrently. Our analysis focused on four main drift diffusion models components: the boundary separation a, the non-decision time t, the drift-rate v and the starting point z. We implement and fit the models using the HDDM library version 0.8.0 (Wiecki, Sofer, and Frank, 2013) and Python 3.6.15. Parameters in HDDM are allowed to “depend on” certain conditions, meaning that the model will estimates separate parameters for each condition. For this analysis, our conditions correspond to the type of trials, i.e., mixed-gamble vs. gain-only, and we design models in which all possible combinations of parameters are varying across types of trials, with the other ones being the same across conditions. In total we test 16 models, namely: none, a, t, v, z, at, av, az, tv, tz, vz, atv, atz, avz, tvz, atvz. Models’ parameters are estimated separately for the low and high anxiety groups based on the GAD-7 cut-off, similarly to the Prospect Theory models described above.

Each model was fitted using HDDM’s MCMC sampler with 4 chains, 1000 burn in samples and 3000 samples. We visually inspected the MCMC traces for good mixing and the autocorrelation plots for low autocorrelations, we also checked the Gelman-Rubin \[\hat{R}\] statistics for convergence (\[\hat{R}\] < 1.1). All models showed good mixing, low autocorrelation and Gelman-Rubin statistics within the desired bounds. Models were compared using the Deviance Information Criterion (DIC) (Ando, 2007) provided by the HDDM library.

We conducted parameter recovery for the HDDMs by simulating 115 agents (high anxiety: 54, low anxiety: 61) using the atvz model. Simulation parameters were sampled from Gaussian distributions with mean and standard deviation coming from the atvz model estimates on our dataset.

### 2.7 Statistical Analysis

We treat self-report questionnaire scores as continuous variables. We extract mean posterior estimates for each subject and we use them as individual computational parameters; these parameters are used to test for correlations with the questionnaire scores. All correlation coefficients are Pearson’s *r*, unless stated otherwise. When parameters are estimated using hierarchical Bayesian model specifications with separate priors, we also report differences in group parameters using the difference between the 95% Highest Density Interval (HDI) of the two group mean estimates. If the resulting HDI does not encompass zero, we conclude that there is a significant difference between the two group means. We also test for between-groups differences using frequentist *t*-tests; all *t*-tests are two-sided with a significance level of *α* = 0.05. Bayesian *t*-tests and correlations have been carried out using JASP (JASP Team, 2023) version 0.17.0. For multiple comparisons tests, we report ‘native’ *p*-values together with the Bonferroni corrected *p*-values. We applied Bonferroni correction for each family of tests we performed: 18 tests related to reaction times (corrected *α* = 0.0027) and 8 tests related to individual choice data (corrected *α* = 0.00625).

## 3 Results

### 3.1 Demographic

Demographic information and self-report questionnaire scores of our sample are reported in Table 1.

**Table 1:** **Sample demographics and questionnaire results**. Values are mean (SD). The last row refers to COVID Score group differences between the vaccinated and unvaccinated groups when the number of vaccines is not included in the score.


GAD-7 SPLIT	All	GAD-7 < 8	GAD-7 ≥ 8	*t*_113_	*p*-VALUE

Women:Men (Sum)	59:56	27:34 (60)	32:22 (55)	–	–

Age in years	39.7 (12.8)	41.9 (13.9)	37.2 (11.2)	1.971	0.051

STAI State Anxiety	40.9 (13.3)	32.6 (8.5)	50.2 (11.5)	–9.376	**<0.001**

STAI Trait Anxiety	48.2 (13.9)	39 (10.1)	58.6 (9.5)	–10.659	**<0.001**

GAD-7	7.3 (5.7)	3.7 (2.3)	13.4 (3.8)	–16.843	**<0.001**

COVID Score	41.1 (13.8)	40 (13.8)	42.4 (13.8)	–0.913	0.363

COVID Score (No #vax)	38.6 (12.7)	37.6 (12.7)	39.8 (12.7)	–0.937	0.351

VACCINATION SPLIT	ALL	VACCINATED	UNVACCINATED	*t*_113_	*p*-VALUE

Women:Men (Sum)	/	37:23 (60)	22:33 (55)	–	–

Age in years	/	40.9 (13.5)	38.4 (11.9)	1.049	0.296

STAI State Anxiety	/	43 (13.8)	38.5 (12.4)	1.819	0.071

STAI Trait Anxiety	/	47.9 (14)	48.5 (13.8)	–0.254	0.799

GAD-7	/	7.8 (5.7)	6.7 (5.7)	1.085	0.279

COVID Score	/	50.9 (8.6)	30.5 (9.9)	11.787	**<0.001**

COVID Score (No #vax)	/	47 (8.4)	29.5 (9.9)	10.267	**<0.001**


To account for the different hierarchical prior specifications tested with our hierarchical Bayesian models, and in line with previous studies examining group differences (Charpentier, Aylward, et al., 2017; Xu et al., 2020), we split our data into low and high anxiety groups and, given our hypothesis about COVID and vaccinations, into unvaccinated and vaccinated groups. A systematic review and meta-analysis study (Plummer et al., 2016) investigating the accuracy of the GAD-7 questionnaire for identifying cases of anxiety disorders found that a cut-off point of GAD-7 ≥ 8 optimises the sensitivity of the questionnaire, without impacting its specificity, compared to the traditional cut-off point of GAD-7 ≥ 10. Throughout the rest of this study, we refer as the low/high anxiety group as the clinically informed split of the data using GAD-7 scores below/above this cut-off (GAD-7 ≥ 8) and as the vaccinated group as subjects who received 1 or more doses of a COVID-19 approved vaccine (high anxiety and vaccinated: *N* = 30; low anxiety and vaccinated: *N* = 30; high anxiety and unvaccinated: *N* = 24; low anxiety and unvaccinated: *N* = 31).

It can be seen from Table 1 that state, trait and GAD-7 anxiety scores are significantly different between groups when splitting the data by the clinically informed GAD-7 cut-off, but there is no significant difference in COVID scores between groups. There is no significant association between gender and the GAD-7 split groups (*χ*^2^(1, *N* = 115) = 2.579, *p* = 0.108). No statistically significant difference were observed between our samples of vaccinated and unvaccinated individuals with respect to state and trait anxiety and GAD-7 scores. However, a statistically significant difference across COVID scores is present – even when the number of vaccination is not included in the score – showing that our questionnaire is able to pick up different attitudes towards COVID between unvaccinated and vaccinated individuals. A significant association between gender and vaccination groups (*χ*^2^(1, *N* = 115) = 5.392, *p* = 0.020) is present.

### 3.2 Choice Data Analysis

There are no correlations between percentages of gamble and state, trait, GAD-7 or COVID questionnaire scores (all *p* > 0.175). We also report no group differences across percentages of gamble across either the low/high anxiety groups or the vaccinated/unvaccinated groups (all *p* > 0.144; see Supp. Inf.). Percentage of gambles across mixed gamble and gain-only trials are not significantly correlated (*r*_115_ = 0.148, *p* = 0.116, BF_10_ = 0.396) indicating that subjects must use different strategies in these different types of trials.

Participants were paid a bonus up to £1, proportional to their final amount of money collected at the end of the gambling trials. The final bonus won by participants was on average £0.37 (£0.08) and this did not correlate with any of our self-report questionnaires (all *p* > 0.183). We also report no significant difference in final bonus between vaccination status (vaccinated: £0.369 (£0.082); unvaccinated £0.371 (£0.077); *t*_113_ = 0.131, *p* = 0.896) or low/high anxiety (low anxiety: £0.318 (£0.087); high anxiety £0.359 (£0.070); *t*_113_ = 1.496, *p* = 0.137).

### 3.3 Risk and Loss Aversion

The Hierarchical Bayesian (HB) fitting method produced more stable results compared to Maximum Likelihood Estimation (MLE) during both parameter recovery and when fitting the participants’ data and for this reason we will only report results for the Hierarchical Bayesian models. The three parameters model (Model 1 – allP) is the best model according to both the AIC, BIC and LOOIC scores and it is the model used for the rest of this analysis (full results in Supp. Inf.).

Our data is best fitted using a single prior for all participants (Table 2), with the model fitted using the clinically informed GAD-7 cut-off priors being a close second.

**Table 2:** **Model 1 LOOIC scores**. Model 1 LOOIC scores for different priors specifications.


	LOOIC

Single prior	**13065.12**

GAD-7 Anxiety priors	13068.45

STAI Trait Anxiety priors	13086.08

Vaccination priors	13083.36

GAD-7 Anxiety and vaccination priors	13101.54

STAI Trait Anxiety and vaccination priors	13086.81


We report the parameters estimated with the single and with the GAD-7 anxiety priors in Table 3. Consistently with the Prospect Theory literature (Kahneman and Tversky, 1979), individuals are on average risk averse (*ρ* < 1) and they value losses twice as much as wins (*λ* ≈ 2).

**Table 3:** Mean (standard deviation) of the fitted parameters of Model 1 using a single prior and a clinically informed GAD-7 cut-off priors.


SINGLE PRIOR	LOSS AVERSION *_λ_*	RISK AVERSION *_ρ_*	INVERSE TEMPERATURE *_μ_*

All participants	2.323 (0.975)	0.933 (0.283)	1.568 (1.526)

Low Anxiety	2.156 (0.971)	0.924 (0.296)	1.829 (1.748)

High Anxiety	2.510 (0.955)	0.943 (0.270)	1.274 (1.178)

GAD-7 PRIOR	LOSS AVERSION *_λ_*	RISK AVERSION *_ρ_*	INVERSE TEMPERATURE *_μ_*

All participants	2.318 (0.967)	0.931 (0.280)	1.573 (1.546)

Low Anxiety	2.125 (0.935)	0.915 (0.296)	1.889 (1.797)

High Anxiety	2.537 (0.966)	0.949 (0.264)	1.217 (1.116)


When using a single hierarchical prior, GAD-7 scores are near significantly correlated to loss aversion (*r*_115_ = 0.168, *p* = 0.072, BF_10_ = 0.576) and inverse temperature (*r*_115_ = –0.166, *p* = 0.075, BF_10_ = 0.555). Risk and loss aversion are not correlated across individuals (*r*_115_ = 0.005, *p* = 0.956, BF_10_ = 0.117), while inverse temperature is negatively correlated with both risk (*r*_115_ = –0.624, *p* < 0.001, BF_10_ > 100) and loss aversion (*r*_115_ = –0.333, *p* < 0.001, BF_10_ = 81.687).

**Figure 2:**
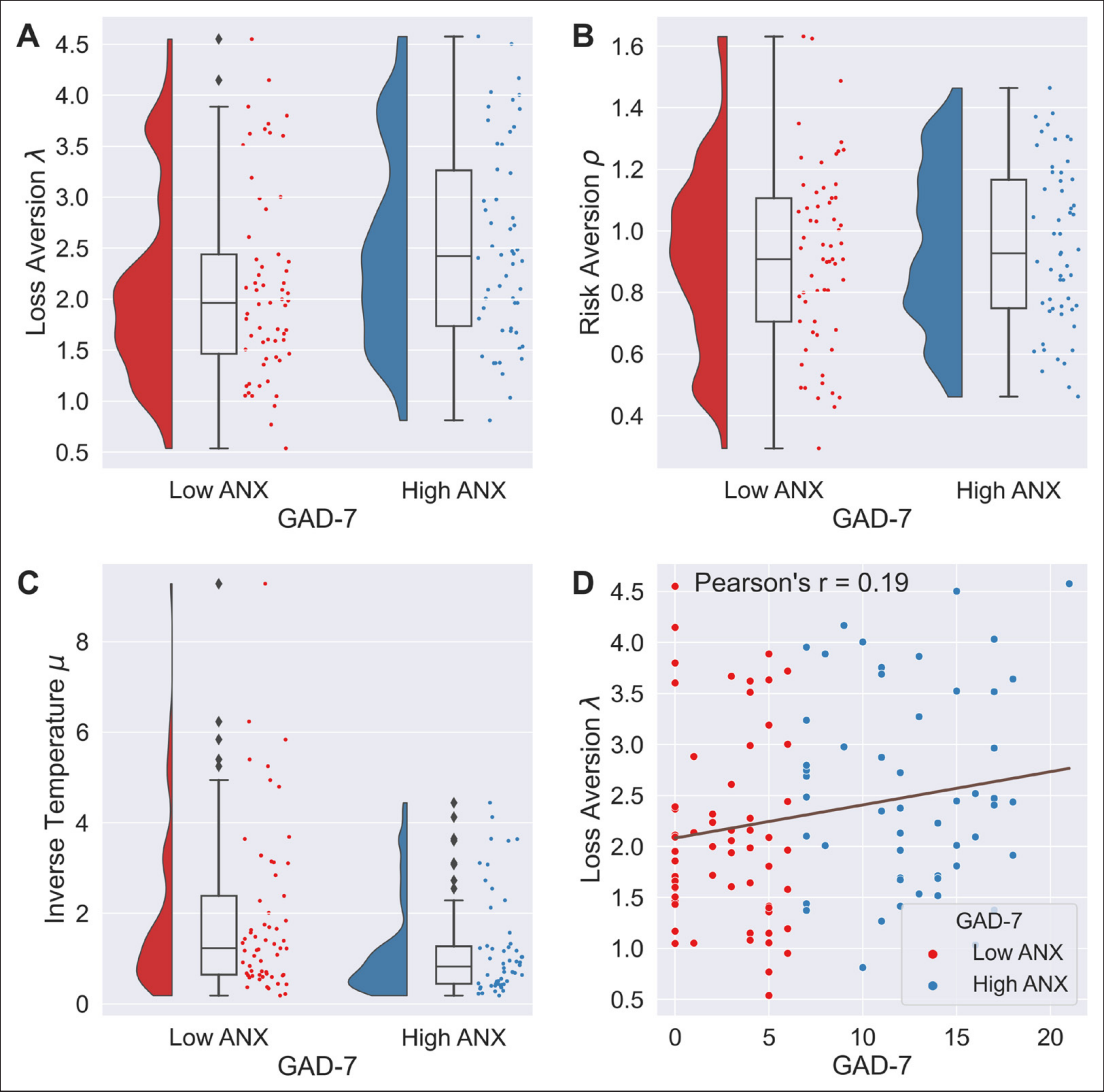
**Model 1 – Parameters estimated under GAD-7 hierarchical priors**. **(A–C)** Parameters distributions for the model. **(D)** Pearson correlation between GAD-7 scores and Loss Aversion parameter.

In our GAD-7 prior model, GAD-7 scores are significantly correlated with loss aversion (*r*_115_ = 0.193, *p* = 0.039, BF_10_ = 0.953, Figure 2(D)) and inverse temperature (*r*_115_ = –0.196, *p* = 0.035, BF_10_ = 1.033), but not risk aversion (*r*_115_ = 0.075, *p* = 0.429, BF_10_ = 0.159). Again, risk and loss aversion are not correlated across individuals (*r*_115_ = 0.004, *p* = 0.963, BF_10_ = 0.117), while inverse temperature is negatively correlated with both risk (*r*_115_ = –0.624, *p* < 0.001, BF_10_ > 100) and loss aversion (*r*_115_ = –0.333, *p* < 0.001, BF_10_ = 79.730). Interestingly, parameters estimates from the single prior specification are near-perfectly correlated with their respective GAD-7 prior estimates (all three parameters: *r*_115_ > 0.99, *p* < 0.001, BF_10_ > 100).

To check whether gender might be driving the correlation between anxiety and loss aversion, we perform two linear regressions of loss aversions estimates on gender and GAD-7 scores in a first model (Model 1) and we include the interaction of gender and GAD-7 scores in a second model (Model 2). We select Model 2 as the best model according to *R*^2^, adjusted *R*^2^ and AIC scores (Model 1: *R*^2^ = 0.070, adjusted *R*^2^ = 0.053, AIC = 317.468, BIC = 328.448; Model 2: *R*^2^ = 0.087, adjusted *R*^2^ = 0.062, AIC = 317.357, BIC = 331.081) while the null model results to be the best model according to BIC scores (null model: AIC = 321.825, BIC = 327.315). The overall regression for Model 2 was statistically significant (*R*^2^ = 0.087, *F*(3; 111) = 3.526, *p* = 0.017) and we found that GAD-7 scores were a significant predictor of loss aversion estimates (*b* = 0.048, *t* = 2.173, *p* = 0.032), while both gender (Male) and the GAD-7 × gender (Male) interaction were not (gender (Male): *b* = 0.011, *t* = 0.036, *p* = 0.971; gender (Male) × GAD-7: *b* = –0.045, *t* = –1.434, *p* = 0.154). We note that in Model 1 gender (Male) is significant (*b* = –0.359, –*t* = 0.180, *p* = 0.026) while GAD-7 scores are not (*b* = 0.026, *t* = 1.629, *p* = 0.106). Based on Model 1 the effect of gender on loss aversion cannot be completely excluded and may deserve further study in future experiments (Georgalos, 2024).

We report significant differences in our GAD-7 prior model in loss aversion (*t*_113_ = 2.317, *p* = 0.022, BF_10_ = 2.156) and inverse temperature (*t*_113_ = 2.371, *p* = 0.019, BF_10_ = 2.407), with the more anxious subjects showing higher values of loss aversion and lower values of inverse temperature. We report no significant difference in risk aversion *ρ* (*t*_113_ = 0.639, *p* = 0.523, BF_10_ = 0.239), see Figure 2(A–C). These group differences are also present when examining the posterior distribution of group means differences between the high and low anxiety groups: loss aversion 95% HDI [0.054, 0.903], risk aversion 95% HDI [–0.089, 0.155], inverse temperature 95% HDI [–0.937, –0.076].

In an effort to replicate the analysis presented in Xu et al. (2020), we fitted a logistic regression model with low/high anxiety (based on the GAD-7 split as above) as the dependent variable and loss *λ* and risk *ρ* aversion parameters as independent variables. In the single prior estimates, loss aversion was a near statistically significant predictor of anxiety group (*z* = 1.922, *p* = 0.055) while risk aversion was not (*z* = 0.343, *p* = 0.732). In the GAD-7 anxiety split estimates, we find that loss aversion was a statistically significant predictor of anxiety group (*z* = 2.242, *p* = 0.025) while risk aversion was not (*z* = 0.641, *p* = 0.522). These results are consistent with Xu et al. (2020), where they found that loss aversion was a significant predictor of anxiety group, while risk aversion was not.

Despite previous research pointing toward differences between low/high STAI Trait anxiety scores, our model fails to capture any significant group difference modulated by trait anxiety. This is surprising given a strong correlation between the STAI Trait Anxiety scores and the GAD-7 scores (*r*_115_ = 0.830, *p* < 0.001, BF_10_ > 10^27^).

### 3.4 COVID Score analysis

Our data do not support any correlation between estimated parameters and COVID score (or the COVID-Past/Future/Generic scores) for either the single or anxiety prior specifications. We report a positive correlation between State Anxiety and COVID score (*r*_115_ = 0.194, *p* = 0.037, BF_10_ = 0.993), State Anxiety and COVID-Future score (*r*_115_ = 0.193, *p* = 0.038, BF_10_ = 0.972) and State Anxiety and COVID-Generic score (*r*_115_ = 0.199, *p* = 0.032, BF_10_ = 1.113). We also find a near significant correlation between State Anxiety and COVID-Past score (*r*_115_ = 0.159, *p* = 0.089, BF_10_ = 0.484).

### 3.5 Reaction Times

Mean reaction times did not correlate with our self-report questionnaires scores (all *p* > 0.220), apart from a near significance negative correlation between GAD-7 scores and mean reaction times in mixed gamble trials in which participants decided not to gamble (*r*_115_ = –0.171, *p* = 0.067, BF_10_ = 0.607). Mean reaction times did not differ across low and high anxiety subjects (low anxiety: 1.254 (0.470) seconds; high anxiety: 1.213 (0.438) s; *t*_113_ = –0.791, *p* = 0.430) or vaccinated and unvaccinated (vaccinated: 1.208 (0.439) s; unvaccinated: 1.264 (0.472) s; *t*_113_ = –1.105, *p* = 0.271). We observe a statistically significant difference in reaction times between low/high anxiety subjects in mixed gamble trials in which participants decided not to gamble: the high anxiety group chooses not to gamble faster compared to the low anxiety group (low anxiety: 1.384 (0.391) s; high anxiety 1.240 (0.245) s; *t*_113_ = –2.326, *p* = 0.021, not significant under a Bonferroni correction with *α* = 0.0027).

### 3.6 Hierarchical Drift Diffusion Models

We find that the atvz model is the best fitting model according to DIC scores. The parameter recovery this model shows good recoverability for the non-decision time t (*r* ∈ [0.65, 0.89]), mixed recoverability for the drift rate v (*r* ∈ [0.34, 0.55]) but poor recoverability for starting point z and boundary separation a (*r* ∈ [0.06–0.5]), likely due to the model fitting procedure introducing correlations between the parameters (see Supp. Inf.). Given the insufficient performance in parameter recovery, we judged the model fitting results unreliable and decided not to elaborate about them here (but see Suppl. Inf. and Discussion).

## 4 Discussion

In this study we found that individuals with high levels of Generalised Anxiety Disorder symptoms show increased loss aversion compared to individuals with lower anxiety levels, as captured by computational parameters of Prospect Theory models estimated using Hierarchical Bayesian methods under clinically informed low and high GAD hierarchical priors specification. No differences in risk aversion were found between the low and high anxiety groups. Our results are consistent with a recent study by Xu et al. (2020), which found increased levels of loss aversion and no differences in risk aversion in individuals with high trait anxiety. Xu et al. used a Prospect Theory model similar to the one used in our study but their task design differs in that they only employed mixed gamble trials in which expected value and variance of the gain/loss amounts were manipulated. Our task, on the other hand, similar to the one used by Charpentier and colleagues (Charpentier, Aylward, et al., 2017), present different types of trials (mixed gamble and gain-only). Notably, our results do not replicate Charpentier et al.’s differences in risk aversion and instead show differences in loss aversion, where they did not find any. This suggests that more work is needed in this field, both to test the robustness of these results and understand the differences observed across different studies. Several competing factors can explain these mixed results. First, our study and Xu et al.’s study were conducted on subclinical populations, whereas Charpentier et al.’s study was conducted on a clinical population, which may indicate possible behavioural differences between subclinical and clinical populations of anxiety. Despite being selected from the general population, however, some of our participants showed clinical levels of generalised anxiety disorders (GAD-7 ≥ 8). Xu et al. and Charpentier et al. only employed STAI trait anxiety scores. Crucially, our study’s results only hold for GAD-7 anxiety scores while we found no relation between loss (nor risk) aversion with STAI Trait anxiety scores. This may highlight the use of largely overlapping but different cognitive mechanisms captured by STAI Trait anxiety scores and GAD-7 scores, despite these being highly correlated both in our study (*r*_115_ = 0.83, *p* < 0.001) and more generally in the literature (Doi et al., 2018). Second, Charpentier et al.’s study (Charpentier, Aylward, et al., 2017) also included an emotional manipulation task which could have had an impact on decision-making that may not be picked up by the models used. A previous study by Charpentier and colleagues (Charpentier, Martino, et al., 2016) found that percentage changes in loss aversion are modulated by emotional cues using the same emotional manipulation in a subclinical population, with low anxious individuals showing greatest increase in loss aversion caused by emotion eliciting stimuli. This study presents some important differences with the current work that may affect direct results comparisons. Namely, in the current work we do not perform any emotional manipulation, our participants reported higher scores of STAI trait anxiety and risk aversion was computed differently compared to both Charpentier’s 2017 study and the current study. Third, our experiment explicitly instructs participants to maximise their final rewards in order to win a larger bonus payment while participants are able to see the result of each gamble (and therefore the amount of money won or lost) at each trial. In Xu et al.’s experiment (Xu et al., 2020), participants were only shown the result of a trial selected at random, with the won/lost amount being added/removed from a fixed amount of money received by the participants before the experiment; in Charpentier et al.’s experiment (Charpentier, Aylward, et al., 2017), participants were shown the results of 10 trials selected at random which were then averaged and either added or removed from a fixed amount of money to reward the participant. This difference in experimental design may explain the overall high values (i.e., reduced risk aversion) that our model fitted for the risk aversion *ρ* parameter (overall: 0.931 (0.281), low anxiety: 0.916 (0.296), high anxiety: 0.949 (0.264)), compared, for example, to a lower mean value of *ρ* reported by Charpentier et al. (overall: 0.713 (0.458), low anxiety: 0.875 (0.537), high anxiety: 0.564 (0.313)). Our study’s explicit instructions to maximise rewards could therefore explain our values of *ρ* close to 1; participants may be motivated to accept more gambles than they normally would in order to increase their in-game scores and their proportional bonus payments, making our incentive less effective for modelling risk aversion than when only one or few trials are shown to the participants (Azrieli, Chambers, and Healy, 2018). Specifically, the current incentive scheme may induce trial-by-trial variation in risk and loss aversion attitude, for example by increasing a participant’s risk and/or loss aversion following a trial in which they lose. It will be important in future work to investigate the effects of different incentive schemes on risk and loss aversion, in the context of anxiety and other psychiatric disorders, for example by testing whether the effects we observe here would replicate if feedback was given through observation of one or a handful of random trials’ outcomes.

We did not find statistically significant differences in reaction times across anxiety levels, apart from the high anxiety group being faster at choosing not to gamble (i.e., avoiding the chance of losing money) in mixed gamble trials. This could be explain in terms of reduced boundary separation, shorter non-decision time, higher drift rate, less biased starting point or a combination of these. Our analysis of reaction times using HDDMs revealed to be inconclusive due to the poor parameter recovery of the models used. This is in part due to the trials structure chosen for this experiment: we intentionally kept the same trials structure employed in Charpentier, Aylward, et al. (2017) in an effort to reproduce the original Prospect Theory results. Future work should focus on testing HDDMs models on tasks specifically designed for them; by, for example, performing extensive parameter recovery and simulations during the task design stage. Future work should also focus on applying more expressive models to our dataset, by for example, incorporating trial-by-trial utilities derived using Prospect Theory into the Drift Diffusion Models (Sheng et al., 2020; Zhao, Walasek, and Bhatia, 2020).

We also highlight how hierarchical Bayesian methods provide better and more reliable parameters’ estimates compared to Maximum Likelihood estimation, as measured in terms of better parameter recovery.

Previous questionnaires-based research (Millroth and Frey, 2021) has highlighted how anxiety and negative individual disposition towards risk and uncertainty have an important role in predicting strong fear responses related to the COVID-19 pandemic. However, in our study, the hypothesis that individual levels of risk and loss aversion could be linked to decision-making in the context of the COVID pandemic was not supported. As previously discussed, the incentive scheme of our task may have motivated our participants to engage in more risk-prone behaviours, compared to a task design in which only one or few trials results are shown to the participants. Furthermore, simple economic decision-making tasks like the one we employed might not be able to capture the intrinsic complexity of decision-making in a multifaceted situation like the COVID pandemic in which economic, health and social factors are all at interplay and more complex naturalistic tasks (Schonberg, Fox, and Poldrack, 2011) may be required to better study these relationships. A recent review about predictors of vaccine hesitancy (Hudson and Montelpare, 2021) includes risk aversion as one of the possible individual difference factors which influence vaccine uptake. Risk aversion could impact vaccine hesitancy in two different and opposite ways. A risk averse individual might value the unknown side effects of a vaccine as riskier than the effects of a known disease and would only get vaccinated due to work or travel requirements. At the same time, an individual might find the dangers posed by a vaccine-preventable disease as riskier than the possible side effects of a vaccine. Loss aversion may also contribute to decisions taken during the pandemic in different ways. For example, workers who would received little protection in case of loss of income due to self isolation following a COVID infection may value the loss of their working hours as more important than the (individual and public) health gains given by self isolating in case of mild COVID symptoms. Therefore, assuming vaccinations and stronger adherence to healthcare guidelines as the risk averse behaviours is a limitation in our study, as some individuals may consider these as more risk- and/or loss-prone actions. Hence, our self-report COVID questionnaire should be expanded to include questions designed to account between these different views.

In conclusion, our results indicate that subjects with high levels of Generalised Anxiety Disorder show increased loss aversion, and no differences in risk aversion, as measured by computational parameters fitted on data from a economic decision-making task. Future computational studies should focus on disentangling the effects of motivation and reward on risky decision-making. These findings may help clinicians in tailoring behavioural interventions in order to overcome decision biases. A previous study (Lorian, Titov, and Grisham, 2012) provides evidence for a reduction of risk-averse behaviours following a cognitive behavioural therapy treatment. Therefore, future work could explore the benefits of including loss aversion as a treatment outcome in similar intervention.

## Data Accessibility Statement

The code and data of this study are available on the Open Science Framework (OSF) at https://osf.io/xqwr5/.

## Additional File

The additional file for this article can be found as follows:

10.5334/cpsy.115.s1 Supplementary Information. Supplementary methods and results including parameter recovery and HDDMs results.
