# Supplementary material for: Risk and Loss Aversion and Attitude to COVID and Vaccines in Anxious Individuals
Source: Comput Psychiatr. 2025 Feb 7;9(1):23–38. doi: 10.5334/cpsy.115 (PMC11804175; doi:10.5334/cpsy.115)
Supplement: Supplementary Information. — Supplementary methods and results including parameter recovery and HDDMs results. [file cpsy-9-1-115-s1.pdf]

Supplementary Information - Risk and Loss Aversion and Attitude to COVID and Vaccines in Anxious Individuals

Filippo Ferrari, Jesse Alexander, Peggy Seriès
Institute for Adaptive and Neural Computation
School of Informatics, University of Edinburgh, United Kingdom

November 2024

A COVID Questionnaire

All items were scored between 1 to 5, we report the questions and the different answers within square brackets. The dots (•) indicate the questions about past behaviours, the asterisks (*) indicate the questions about future behaviours and the dashes (-) indicate the questions about general attitude towards COVID. We group these questions in COVID-Past (•), COVID-Future (*) and COVID-General (-) scores.

• How many COVID vaccinations have you had? [0, 1, 2, 3, 4+]

* Would you get another booster dose if offered? [Very Unlikely, ..., Very Likely]

• What type of mask did you usually wear throughout the pandemic? [No Mask, Scarf/Bandana/Other, Reusable Fabric Mask, Surgical/Disposable Mask, Respirator (N99/N95)]

* What type of mask would you wear more often in case of a new surge in COVID cases? [No Mask, Scarf/Bandana/Other, Reusable Fabric Mask, Surgical/Disposable Mask, Respirator (N99/N95)]

• How anxious were you about coronavirus at the beginning of the pandemic in March 2020? [Not at all Anxious, ..., Extremely Anxious]

* How anxious are you about a new increase in COVID cases in the upcoming Winter? [Not at all Anxious, ..., Extremely Anxious]

• How closely have you followed the government rules and guidelines during the pandemic? [I have not followed any of the rules, ..., I have followed all of the rules]

* In case of a hypothetical new pandemic how closely would you follow new government rules and guidelines? [I would not follow any of the rules, ..., I would follow all of the rules]

- I think Coronavirus vaccinations should be mandatory. [Strongly Disagree, ..., Strongly Agree]

- During the pandemic I wore a mask and social distanced as often as I could. [Strongly Disagree, ..., Strongly Agree]

- I think that Coronavirus restrictions introduced in 2020 were necessary and important. [Strongly Disagree, ..., Strongly Agree]

- I am worried or have been worried about coronavirus affecting my health. [Strongly Disagree, ..., Strongly Agree]

- I am worried or have been worried about coronavirus affecting the health of my friends and family. [Strongly Disagree, ..., Strongly Agree]

B Indifference Points

The 40 practice trials consisted of 24 for mixed-gamble and 16 gain-only trials. A double staircase procedure adjusted the gamble expected value to each participant's indifference point (IP, i.e. the expected value at which the participant gambles 50% of the time). This procedure was adapted from Charpentier, Martino, et al. (2016) and Charpentier, Aylward, et al. (2017) and consisted of 12 and 8 sets of 2 trials for mixed-gamble and gain-only trials, respectively. In the first trial the expected value of the gamble was high, and in the second the expected value of the gamble was low. If the gamble with high expected value was accepted, the low IP for the participant was decreased by 0.5, and, if the low expected value gamble was accepted, the high IP increased by 0.5. In case the low and high IPs did not converge to the same value by the end of the procedure, the average of the two IPs was taken as the participant's IP. This procedure was separate for mixed-gamble and gain-only trials, resulting in separate IPs for the two types of trials.

IPs for mixed-gamble trials were used to compute 7×7 gain-loss matrices of mixed-gamble gambles with fixed loss values of $[-2, -4, -6, -8, -10]$ centered on each participant's IP, i.e. the values on the diagonal are $\text{IP} = 0.5 \cdot \text{gain} + 0.5 \cdot \text{loss}$. Similarly, 5×5 gain-sure matrices for gain-only trials were computed using fixed sure values of $[3, 4, 5, 6, 7]$.

Overall, IPs for mixed-gamble trials had mean 4.465 and standard deviation 1.306. IPs for gain-only trials had mean 1.417 and standard deviation 1.956. IPs for mixed-gamble trials were not significantly correlated with trait anxiety ($r_{115} = 0.089$, $p = 0.344$), state anxiety ($r_{115} = 0.037$, $p = 0.693$), GAD-7 ($r_{115} = 0.116$, $p = 0.219$) or COVID Score ($r_{115} = 0.334$, $p = -0.091$). IPs for gain-only trials were not significantly correlated with trait anxiety ($r_{115} = 0.021$, $p = 0.820$), state anxiety ($r_{115} = -0.002$, $p = 0.982$), GAD-7 ($r_{115} = 0.013$, $p = 0.890$) or COVID Score ($r_{115} = -0.045$, $p = 0.629$).

C Exclusion Criteria

Different types of exclusion criteria have been used. During the gambling part of the experiment, 4 trials were used as attention checks. In these trials the subjects had to choose between a win of £0 and a loss in the range £[-30,-5] together with a sure option of £0. We discarded participants who chose the gambling option for 2 or more of these 4 attention checks. Another attention check was included in the questionnaire part of the experiment. We removed 22 participants who failed 2 or more gambling attention checks whereas no participant failed the questionnaire attention check. Four participants were removed due to incomplete demographic information.

We also removed participants who did not show any differences in percentage of gambling as a function of the trials' expected values. We remove participants who have a difference in average percentage of gamble lower than 5% between the upper half of expected values and the lower half of expected values. Participants should be able to adapt their gambling to the different expected values of the trials (i.e., higher expected values should lead to higher percentage of gambles). An example of this can be seen in fig. 1. We removed 22 participants using this criteria.

In total we conducted the analysis on 115 participants.

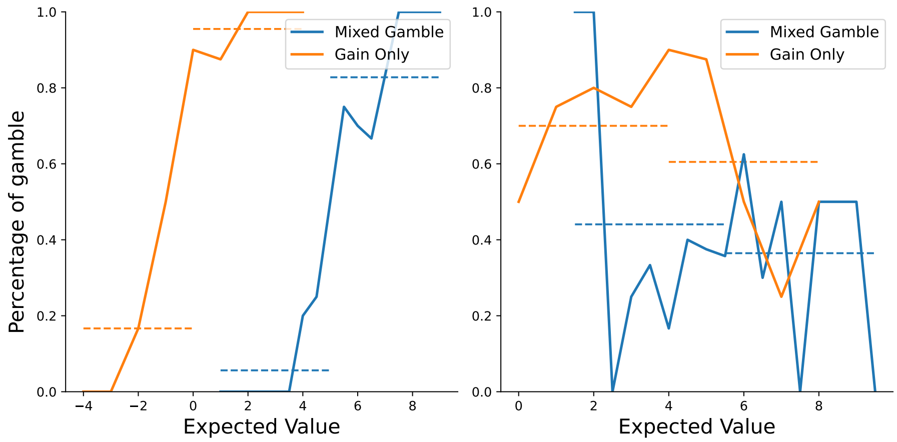

Figure 1: On the left, a participants who correctly adapted their choices to the expected value of the trials. On the right, a participant who incorrectly reduces their percentage of gambles for gambles with higher expected values. The participant on the right was removed from further analysis.

D Parameter Recovery and Model Comparison

Parameter recovery was conducted simulating 100 subjects for each combination of parameters/indifference points. Parameters were sampled from Gaussian distributions using the fitted parameters reported by Charpentier, Aylward, et al. (2017) (reported here in table 1). Indifference points were sampled from Gaussian distributions with SD = 2 and means as follows: High IP mixed gamble = 8, High IP gain only = 4, Low IP mixed gamble = 2, Low IP gain only = 1.

	Low Anxiety	High Anxiety
λ	2.067 (0.752)	2.013 (0.625)
ρ	0.875 (0.537)	0.713 (0.458)
μ	3.857 (4.502)	5.119 (4.124)

Table 1: Parameters from Charpentier, Aylward, et al. (2017) used for the parameter recovery. Mean (Standard deviation).

The results for the parameter recovery are reported in table 2. Due to outliers in the MLE fitting of the data we report the more robust Spearman correlation coefficients.

	MLE			HB		
	λ	ρ	μ	λ	ρ	μ
148 trials						
High Anxiety / Low IP	0.82	0.88	0.88	0.91	0.96	0.90
High Anxiety / High IP	0.71	0.93	0.87	0.92	0.96	0.84
Low Anxiety / Low IP	0.80	0.88	0.91	0.95	0.97	0.79
Low Anxiety / High IP	0.84	0.90	0.84	0.89	0.94	0.75
222 trials						
High Anxiety / Low IP	0.82	0.87	0.95	0.93	0.99	0.94
High Anxiety / High IP	0.77	0.91	0.78	0.89	0.97	0.92
Low Anxiety / Low IP	0.77	0.90	0.93	0.95	0.98	0.92
Low Anxiety / High IP	0.64	0.87	0.90	0.87	0.95	0.74
296 trials						
High Anxiety / Low IP	0.85	0.97	0.94	0.94	0.97	0.97
High Anxiety / High IP	0.82	0.96	0.87	0.95	0.98	0.93
Low Anxiety / Low IP	0.81	0.93	0.89	0.98	0.99	0.90
Low Anxiety / High IP	0.89	0.92	0.87	0.86	0.96	0.82

Table 2: Parameter recovery for 148, 222 and 296 trials. We report Spearman Correlation Coefficients. IP = Indifference Point. MLE is Maximum Likelihood Estimation, HB is Hierarchical Bayesian.

Table 3: AIC, BIC and LOOIC scores for the three models. Lower is better.

	MLE		HB
	AIC	BIC	LOOIC
Model 1 (allP)	**46084.59**	**13768.63**	**13065.12**
Model 2 (noRA)	54758.99	21868.35	14234.04
Model 3 (noLA)	52286.11	19395.47	18819.73

E Choice Data Analysis

Table 4: Probability of gamble during trials. Here p(gamble-mixed) and p(gamble-gain) refers to the probability of gambling in, respectively, mixed gamble and gain only trials.

	p(gamble)	p(gamble-mixed)	p(gamble-gain)
All participants	0.587	0.576	0.609
Low Anxiety	0.597	0.603	0.586
High Anxiety	0.577	0.546	0.636
Vaccinated	0.578	0.553	0.628
Unvaccinated	0.597	0.601	0.589

F Model 1

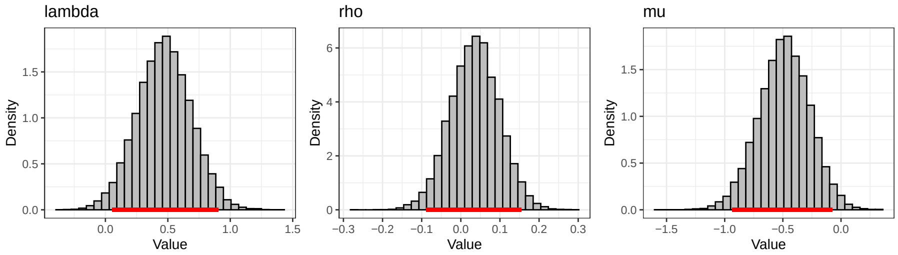

Figure 2: 95% HDI of the group mean differences for the loss aversion, risk aversion and inverse temperature parameters of Model 1. Parameters estimated using the GAD-7 anxiety prior.

G HDDMs - Parameter recovery

`atvz` model	`a`	`t`	`v`	`z`
High Anxiety - Mixed-gamble	0.50	0.85	0.55	0.24
High Anxiety - Gain-only	0.06	0.65	0.33	0.41
Low Anxiety - Mixed-gamble	0.27	0.89	0.51	0.13
Low Anxiety - Gain-only	0.21	0.85	0.50	0.34

Table 5: Parameter recovery for the `atvz` model. We report Pearsons' r correlations.

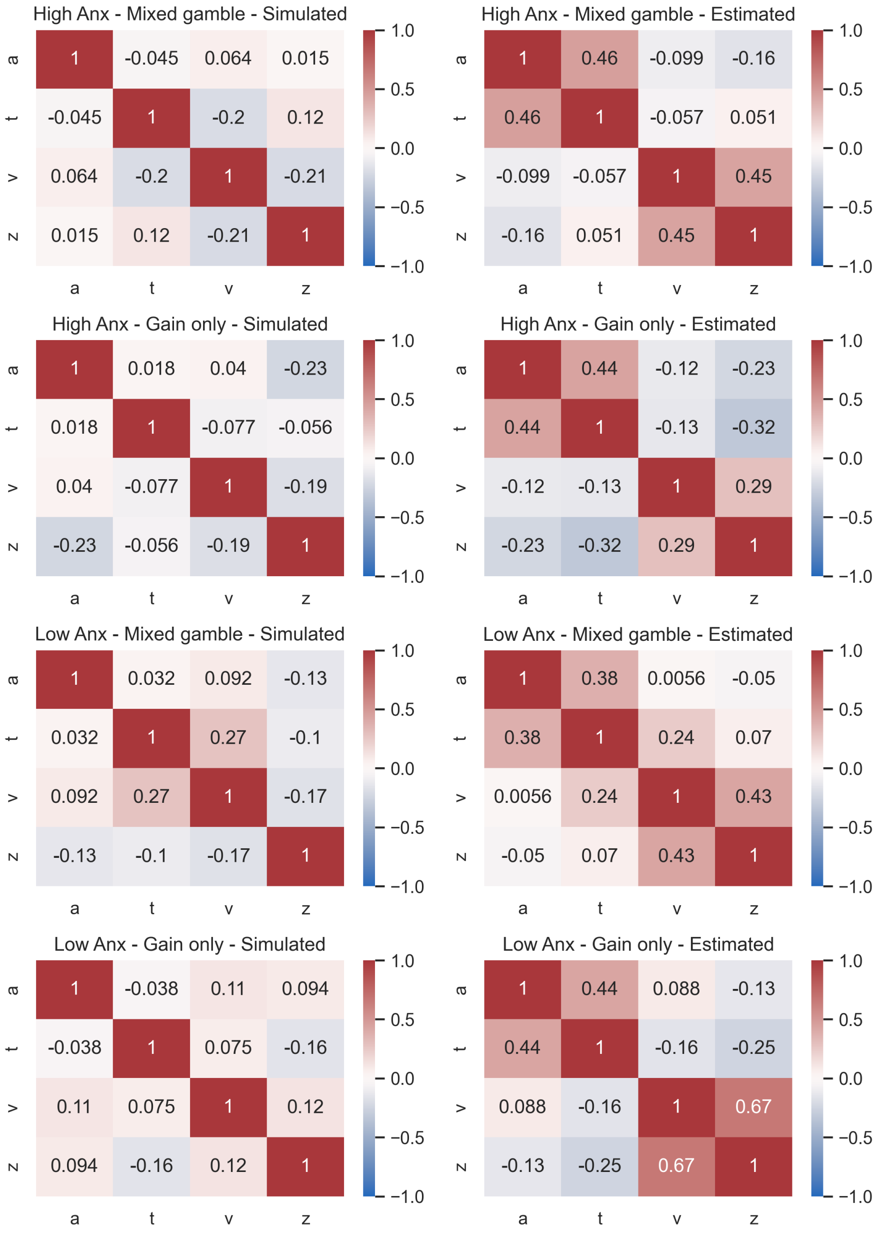

Figure 3: Correlations between parameters in the parameter recovery of the `atvz` model (best fitting model).

H HDDMs - Extra results

The model fitting results of the HDDMs are reported in table 6.

Model	Low Anx DIC	High Anx DIC	Total DIC	Rank
none	17071.3	15590.0	32661.3	16
a	16958.1	15468.9	32427.0	15
t	16182.5	15094.7	31277.2	10
v	16273.1	15183.7	31456.8	12
z	16453.7	15327.6	31781.3	14
at	16122.5	15045.0	31167.4	8
av	16238.9	15069.6	31308.5	11
az	16439.5	15240.9	31680.4	13
tv	15492.1	14699.7	30191.7	4
tz	15830.0	14890.8	30720.8	6
vz	16120.3	15079.1	31199.4	9
atv	15400.0	14660.7	30060.7	2
atz	15738.3	14858.0	30596.2	5
avz	16105.2	15002.5	31107.8	7
tvz	15450.8	14660.6	30111.3	3
atvz	15338.0	14601.5	**29939.6**	1

Table 6: Model fitting results based on Deviance Information Criterion (DIC), lower is better. a is boundary separation, t is non-decision time, v is drift-rate, z is starting point.

Table 7: HDDM group differences for winning atvz model. Group differences between the low and high anxiety groups in the atvz model. a is boundary separation, t is non-decision time, v is drift-rate, z is starting point.

	t-test (p-value)		95% HDI	
	mixed-gamble	gain-only	mixed-gamble	gain-only
a	1.970 (0.051)	-0.520 (0.604)	[-0.168, 0.001]	[-0.085, 0.102]
t	0.724 (0.470)	1.651 (0.101)	[-0.100, 0.042]	[-0.135, 0.011]
v	1.414 (0.160)	-1.950 (0.053)	[-0.410, 0.072]	[-0.048, 0.452]
z	0.748 (0.456)	1.761 (0.081)	[-0.033, 0.021]	[-0.048, 0.011]

No significant difference can be found between groups for any of the parameters of the atvz winning model (table 7), with the exception of a near significant difference in boundary separation a in mixed-gamble trials and its 95% HDI difference very close to not encompass zero. High anxiety subjects show reduced boundary separation (high anxiety $\bar{a} = 1.514$; low anxiety $\bar{a} = 1.596$) in mixed-gamble trials, a result which is difficult to interpret in terms of risk and loss

aversion when presented on its own. In fact, the boundary separation `a` is is significantly correlated with the drift rate `v` ($r_{115} = 0.403$, $p < 0.001$), which is in turn significantly correlated with the starting point `z` ($r_{115} = 0.330$, $p < 0.001$), making the overall interpretability of these results quite difficult. Subjects' estimates for these parameters do not correlate with our questionnaires of interest (STAI-State, STAI-Trait, GAD-7, COVID Score; all correlations $p > 0.113$).

The posterior distributions of the estimated parameters for the `atvz` model are shown in fig. 4.

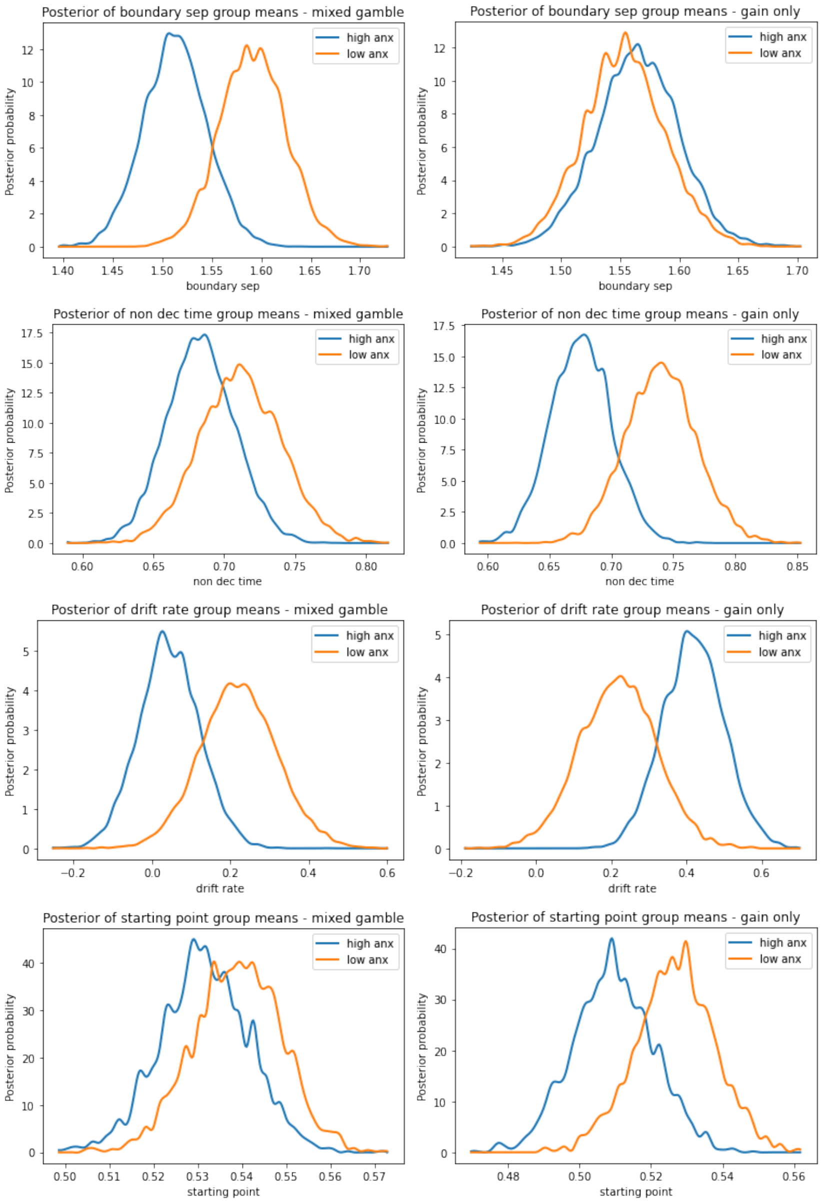

Figure 4: Posteriors distributions of the parameters fitted using the `atvz` model. Posteriors for the mixed-gamble trials are on the left.

References

Charpentier, Caroline J., Jessica Aylward, et al. (June 2017). “Enhanced Risk Aversion, But Not Loss Aversion, in Unmedicated Pathological Anxiety”. In: *Biological Psychiatry* 81.12, pp. 1014–1022. ISSN: 00063223. DOI: 10.1016/j.biopsych.2016.12.010.

Charpentier, Caroline J., Benedetto De Martino, et al. (Apr. 2016). “Emotion-Induced Loss Aversion and Striatal-Amygdala Coupling in Low-Anxious Individuals”. In: *Social Cognitive and Affective Neuroscience* 11.4, pp. 569–579. ISSN: 1749-5024, 1749-5016. DOI: 10.1093/scan/nsv139.
